# NO-Rich Diet for Lifestyle-Related Diseases

**DOI:** 10.3390/nu7064911

**Published:** 2015-06-17

**Authors:** Jun Kobayashi, Kazuo Ohtake, Hiroyuki Uchida

**Affiliations:** Division of Pathophysiology, Department of Clinical Dietetics and Human Nutrition, Faculty of Pharmaceutical Science, Josai University, Saitama 350-0295, Japan; E-Mails: kazuo@josai.ac.jp (K.O.); mrhiro@josai.ac.jp (H.U.)

**Keywords:** lifestyle-related disease, nitric oxide (NO), nitrate, nitrite, insulin resistance, ischemia/reperfusion injury, chronic obstructive pulmonary disease (COPD), osteoporosis, cancer

## Abstract

Decreased nitric oxide (NO) availability due to obesity and endothelial dysfunction might be causally related to the development of lifestyle-related diseases such as insulin resistance, ischemic heart disease, and hypertension. In such situations, instead of impaired NO synthase (NOS)-dependent NO generation, the entero-salivary nitrate-nitrite-NO pathway might serve as a backup system for NO generation by transmitting NO activities in the various molecular forms including NO and protein *S*-nitrosothiols. Recently accumulated evidence has demonstrated that dietary intake of fruits and vegetables rich in nitrate/nitrite is an inexpensive and easily-practicable way to prevent insulin resistance and vascular endothelial dysfunction by increasing the NO availability; a NO-rich diet may also prevent other lifestyle-related diseases, including osteoporosis, chronic obstructive pulmonary disease (COPD), and cancer. This review provides an overview of our current knowledge of NO generation through the entero-salivary pathway and discusses its safety and preventive effects on lifestyle-related diseases.

## 1. Introduction

Health problems, such as insulin resistance, cardiovascular disease, osteoporosis, and cancer share some common risk factors, including unhealthy and excessive nutrition, a lack of physical activity, smoking and heavy drinking [1]. So-called lifestyle-related diseases are now the leading causes of mortality and morbidity in developed countries [2]. Healthy diets and exercise training are low-cost and easily-practicable lifestyle changes to be recommended for patients with these conditions before starting pharmacological therapy. Recent prospective and epidemiologic studies have shown that among the various foods, green leafy vegetables are undoubtedly protective against coronary heart disease, hypertension [3,4,5,6], and ischemic stroke [7]. This may be because vegetables and fruits rich in nitrate can provide a physiological substrate for reduction to form nitrite and nitric oxide (NO). The beneficial effects of these foods on the diseases resulting from circulatory disturbances are attributed to the cyclic guanosine monophosphate (cGMP)-dependent actions of NO, including vasodilation and vascular endothelial protection from platelet aggregation and leukocyte adhesion [8]. However, in addition to these classical functions of NO, recent studies have indicated novel functions for NO through cGMP-independent and protein *S*-nitrosylation-dependent intracellular signaling pathways [9]. *S*-nitrosylation is associated with the activation of transcription factors, the regulation of a number of signal transduction molecules [10] and redox protein modification [11], mitochondrial functions [12,13], and cell apoptosis [14], which could explain how the dietary nitrate exerts preventive effects against the development of lifestyle-related metabolic, inflammatory, and proliferative disorders. This review provides an overview of our current knowledge of NO production through the dietary nitrate-nitrite-NO pathway and its physiological aspect, then discusses the safety and efficacy of dietary nitrate, as well as its preventive effects on lifestyle-related diseases.

## 2. The Dietary Nitrate-nitrite-NO Pathway and Its Physiological Aspect

**Figure 1:**
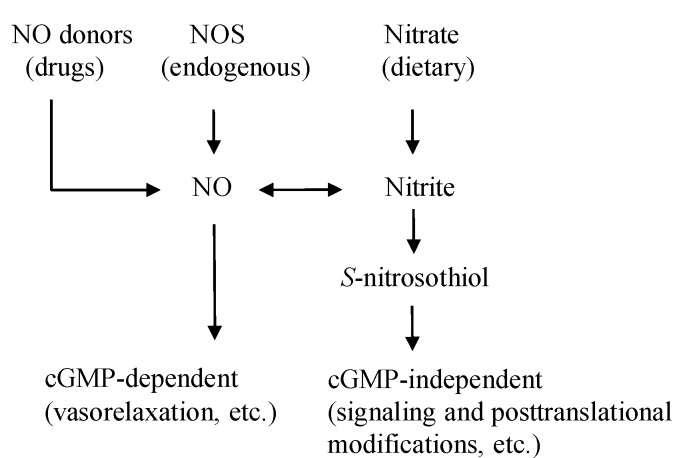
The NO pathways. NO is originated from the diet, drugs, and the endogenous NOS enzyme. The activities of NO are exerted through cGMP-dependent and independent ways. NO donor drugs, such as nitroglycerin and sodium nitroprusside, directly release NO. NOS endogenously generates NO, which is linked to the cGMP-dependent functions of NO. Dietary nitrate/nitrite produce NO and *S*-nitrosothiol, mainly for cGMP-independent functions. NO: nitric oxide, NOS: NO synthase, cGMP: cyclic guanosine monophosphate.

**Figure 2:**
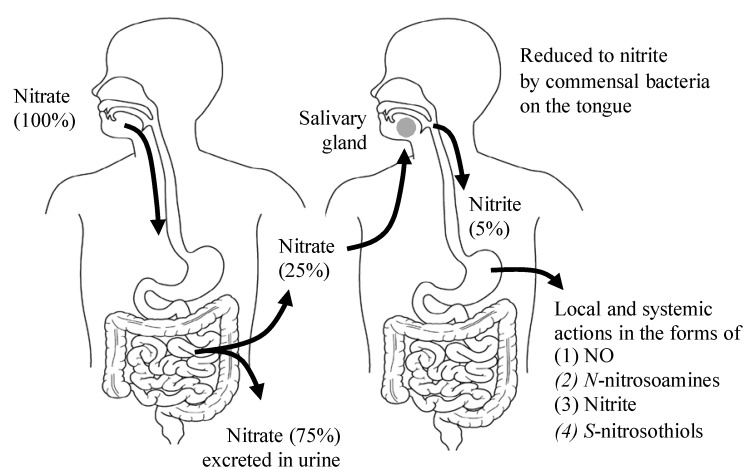
The entero-salivary nitrate-nitrite-NO pathway. Twenty-five percent of the ingested dietary nitrate is recycled to the saliva, and 20% of the nitrate in saliva is converted to nitrite by oral commensal bacteria. Approximately 5% of the originally ingested nitrate is swallowed into the stomach, and provides for NO activities in various forms. (1) NO for local vasodilation, mucus formation, and antimicrobial activity; (2) *N*-nitrosoamines for local carcinogenesis; (3) Nitrite for nitrite pool and transnitrosylation in the peripheral tissues; (4) *S*-nitrosothiols for transnitrosylation in the peripheral tissues.

In addition to endogenous NO generation through the l-arginine-NO synthase (NOS) pathway, NO is also generated through the NOS-independent nitrate-nitrite-NO pathway [15] (Figure 1). Green leafy vegetables such as lettuce, spinach and beetroot all contain high concentrations of nitrate [16] (Table 1). Vegetables account for 60%–80% of the daily nitrate intake in a Western diet [17], and substantial elevations in the plasma nitrite levels can occur through increasing the dietary nitrate intake [18]. One serving of such a vegetable contains more nitrate than what is endogenously generated by NOS isoforms during a full day in humans [19]. As shown in Figure 2, the ingested dietary nitrate is absorbed in the upper gastrointestinal tract and reaches the peak plasma level around 30–60 min after ingestion [20] (Table 2). Approximately 25% of the absorbed nitrate is delivered to the salivary gland and secreted into saliva in the oral cavity [21], which is then reduced to nitrite by commensal anaerobic bacteria on the tongue [22]. In the acidic environment of the stomach, part of the swallowed nitrite is immediately protonated to nitrous acid (NO_2_^−^ + H^+^ → HNO_2_), then decomposed to form a variety of nitrogen oxides such as NO, nitrogen dioxides (NO_2_), and dinitrogen trioxide (N_2_O_3_) (2 HNO_2_ → N_2_O_3_ + H_2_O, N_2_O_3_ → NO + NO_2_) [23]. These nitrogen oxides form additional bioactive adducts, such as *S*-nitrosothiols and *N*-nitrosoamines, following reactions with protein thiols and amines, respectively, in the dietary products. In particular, the NO production in the stomach is greatly enhanced in the presence of dietary polyphenols [24] and ascorbic acid [15], whereas because of its lower stability and shorter half-life relative to *S*-nitrosothiols, the released NO in the stomach is thought to locally contribute to increasing the gastric mucosal blood flow and mucous thickness to ensure the normal gastric physiology, and serves as the first-line host defense against swallowed pathogens [25,26]. However, some of the nitrite escapes the protonation in the acidic milieu of the stomach and enters the systemic circulation, and then reaches the peripheral organs, including skeletal muscles, where it acts in an endocrine manner to exert NO-like activity [18] (Figure 2). Because the plasma levels of nitrite are highly dependent on the amount of salivary nitrate and its reduction to nitrite, the use of an antibacterial mouthwash [27] and frequent spitting of saliva consequently decrease the plasma levels of nitrite [20].

**Table 1:** Nitrate and nitrite contents of food products.

Food product	Nitrate concentration (mg/100 g)		Nitrite concentration (mg/100 g)		Reference
	Mean	Range	Mean	Range	
Beets	275.6	168–359	1.00	0.21–2.98	[16]
Spinach	233.3	53.5–366	0.70	0.0–1.29	
Radishes	168.0	76.4–250	0.01	0.0–0.1	
Celery	154.4	31.6–332	0.16	0.0–0.52	
Lettuce	85.0	7.9–217.1	0.06	0.001–0.97	
Iceberg lettuce	78.6	34.7–108	0.02	0.0–0.17	
Mushroom	59.0	1.9–8.5	0.80	0.0–3.8	
Cabbage	57.3	19.3–97.6	0.24	0.0–1.26	
Broccoli	39.4	2.9–114	0.06	0.001–0.95	
Green beans	38.6	16.5–61.1	0.05	0.0–0.25	
Strawberries	17.3	10.5–29.3	0.20	0.0–0.71	
Banana	13.7	8.8–21.4	0.21	0.0–0.95	
Green pepper	3.3	0.8–5.5	0.04	0.0–0.3	
Spinach	741.0	-	0.02	-	[28]
Mustard greens	116.0	-	0.003	-	
Salad mix	82.1	-	0.13	-	
Cole slaw	55.9	-	0.07	-	
Broccoli	39.5	-	0.07	-	
Tomato	39.2	-	0.03	-	
Vegetable soup	20.9	-	0.001	-	
Hot dog	9.0	-	0.05	-	
Bacon	5.5	-	0.38	-	
Banana	4.5	-	0.009	-	
Pork tenderloin	3.3	-	0.0	-	
Bacon nitrite-free	3.0	-	0.68	-	
French fries	2.0	-	0.17	-	
Ham	0.9	-	0.89	-	
Fruit mix	0.9	-	0.08	-	
Orange	0.8	-	0.02	-	
Apple sauce	0.3	-	0.008	-	
Ketchup	0.1	-	0.13	-	
Carrots	0.1	-	0.006	-	
	**Nitrate concentration (mg/L)**		**Nitrite concentration (mg/L)**		
Carrot juice	27.55	-	0.036	-	
Vegetable juice *	26.17	-	0.092	-	
Pomegranate juice	12.93	-	0.069	-	
Cranberry juice	9.12	-	0.145	-	
Acai juice	0.56	-	0.013	-	
Green tea	0.23	-	0.007	-	

* V8; Campbell Soup Co (Camden, NJ, USA); Table is reproduced from the reference [16] and [28].

**Table 2:** Saliva and plasma levels of nitrite, nitrate, and *S*-nitrosothiol before and 30 min after oral administration of sodium nitrate (10 mg/kg) in healthy volunteers.

		0 Min	30 Min
Saliva	Nitrite (μM)	104 ± 21	713 ± 150
	Nitrate (mM)	0.19 ± 0.03	8.2 ± 1
	*S*-NO (nM)	25 ± 9.8	297
Plasma	Nitrite (μM)	123 ± 19	229 ± 46
	Nitrate (mM)	30 ± 4	432 ± 44
	*S*-NO (nM)	6.3 ± 1.4	No significant change

Data from reference 20, *S*-NO: *S*-nitrosothiol.

The plasma nitrite which reaches peripheral tissues is stored in various organs. Although there have been few reports dealing with the tissue levels of nitrate/nitrite following dietary nitrate supplementation in humans, animal studies show that dietary nitrate certainly increases the tissue levels of nitrate/nitrite following increase in the plasma levels of nitrate/nitrite (Table 3), which accordingly exerts therapeutic efficacy for animal models of various disease conditions. Interestingly, while acute dietary nitrate intake increases the plasma levels of nitrite in rodents and humans [10,20], chronic dietary nitrate intake does not always increase the plasma and tissue levels of nitrite, but increases the tissue levels of nitrate and *S*-nitrosylated products (Table 3). Although the mechanism underlying this finding is yet to be clarified, there might be some redox equilibrium of nitrate-nitrite-NO after chronic dietary nitrate intake, resulting in oxidation or reduction of the tissue nitrite to form nitrate or *S*-nitrosylated species, respectively. On the other hand, animal models chronically fed a diet deficient in nitrate/nitrite exhibit significantly diminished plasma and tissue levels of nitrate/nitrite, resulting in increased ischemia-reperfusion injuries in heart and liver compared with the animal models fed a normal diet [29,30]. These results suggest that dietary nitrate intake is important in the maintenance of steady-state tissue levels of nitrate/nitrite for NO-mediated cytoprotection.

Various enzyme/protein-dependent reductions to NO have been proposed under physiological and pathological conditions, which include deoxyhemoglobin [31], deoxymyoglobin in the skeletal, vascular [32], and cardiac muscles [33], xanthine oxidase in endothelial cells, aldehyde oxidase, aldehyde dehydrogenase 2 [34], cytochrome P-450, and mitochondrial nitrite reductases (such as mitochondrial electron transport complexes) in all cells. In contrast to NOS-dependent NO production, which requires molecular oxygen, this nitrite reduction to NO is enhanced under hypoxic and acidic conditions. Because the nitrite-reducing factors are rich in skeletal muscles, tissue hypoxia during submaximal exercise kinetically favors nitrite reduction to NO, thus providing an alternative NO source for vascular dilation and efficient O_2_ consumption in the working muscles [35,36].

**Table 3:** The effects of chronic dietary nitrate supplementation on tissue levels of nitrate/nitrite and therapeutic efficacy for experimental animal models.

Animal Model	Dietary Nitrate	Tissues	Effects of Dietary Nitrate	References
Uninephrectomized hypertension rat with high-salt diet.	Diets with 0.1 mM and 1 mM nitrate/kg/day for 8–11 weeks.	Kidney Heart Liver	Increase in plasma and tissue levels of nitrate and tissue levels of nitrosylation products. Reduction of oxidative stress and attenuation of renal injury, hypertension, cardiac hypertrophy and fibrosis.	[37]
C57BLK6 male mice with hypoxia-induced pulmonary hypertension.	0.6 mM, 15 mM, and 45 mM nitrate/L in drinking water for 3 weeks.	Lung	Increase in plasma and lung levels of nitrite and cGMP. Reduction of right ventricular pressure and hypertrophy, and pulmonary vascular remodeling.	[38]
Male Wistar rat with hypoxic heart damage.	0.7 mM/L nitrate in drinking water for 2 weeks.	Heart	Increase in plasma levels of nitrate and tissue levels of nitrite. Alleviation of metabolic abnormalities in the hypoxic heart. Improvement of myocardial energetics.	[39]

**Figure 3:**
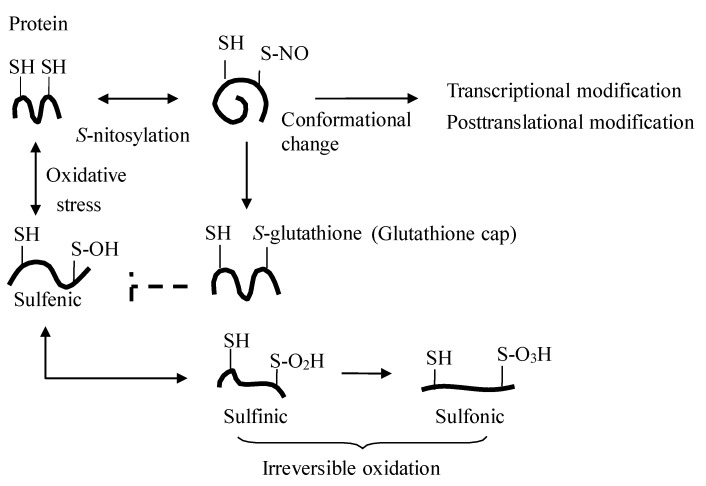
Protein *S*-nitrosylation. *S*-nitrosylation of protein elicits its regulatory effect by adding the NO moiety on the active thiol (SH of cysteine residue) of the protein (e.g., transcriptional factors and enzymes), and cell protection by the subsequent posttranslational addition of glutathione to the protein thiols (so-called glutathione cap), which shields the cysteine residues from further irreversible protein oxidation [11].

Although elevated plasma levels of nitrite certainly affect the cGMP production in systemic organs, providing an important signaling role in mammalian biology [10], dietary nitrate/nitrite also transmit biological signals via cGMP-independent mechanisms, such as transnitrosylation, a posttranslational modification analogous to phosphorylation, in order to regulate the protein function [40] (Figure 1 and Figure 3). Here, the questions are raised. What is the carrier of NO activity to the peripheral organs, *S*-nitrosothiol, NO itself, or nitrite, and also, how does this carrier transnitrosylate in the peripheral organs? Lundberg *et al.*, showed that oral intake of sodium nitrate (10 mg/kg) in healthy volunteers significantly increased plasma levels of nitrite, but did not increase *S*-nitrosothiol in plasma [20] (Table 2). In addition, Bryan *et al.*, showed that protein *S*-nitrosylation in organs following intraperitoneal nitrite injection to rat, could not be inhibited by NO scavenging with carboxy-2-phenyl-4,4,5,5-tetramethylimidazolin-1-oxyl-3-oxide (cPTIO), suggesting that the nitrite-mediated transnitrosylation in the organs might occur mainly directly through nitrite rather than through either circulating *S*-nitrosothiol or NO itself [10].

Bryan *et al.*, also showed that the increase in plasma nitrite within the physiological concentration rage of 0.2–2 μM after nitrite administration (plasma concentration of nitrite far lower than those required for vasodilation) enhanced *S*-nitrosothiol in the organs (heart, kidney, liver, lung, and aorta) with the subsequent modulation of signaling and gene expressions of cGMP, cytochrome P-450, heat shock protein 70, and heme oxygenase-1 in these organs. Interestingly, they also showed that a switch of the standard chow to the low nitrate/nitrite diet for two days in rats decreased nitrite levels substantially in all tissues and represented changes of the signaling and gene expressions in a direction opposite to those found with nitrite administration [10].

These observations indicate that the nitrite-induced transnitrosylation in organs might be an alternative *in vivo* nitrite signaling for the mammalian biology including protection of protein thiols from irreversible oxidation, transcriptional modulation, and posttranslational regulation of most classes of proteins present in all cells [9] (Figure 3), and also that changes in plasma nitrite levels even within the physiological ranges (e.g., postprandial and fasting) can affect tissue levels of *S*-nitrosothiol and subsequent cellular biology.

## 3. Safety and Efficacy of Dietary Nitrate

Very high concentrations of nitrate in drinking water may cause methemoglobinemia in infants (blue baby syndrome) [41]. In the 1940s, Comly first reported cases of cyanotic infants who received formula prepared with well water containing a high nitrate content [42]. Based on the subsequent analyses of the infantile cases of methemoglobinemia, the US Environmental Protection Agency (EPA) set a Maximum Contaminant Level (MCL) for nitrate of 44 mg/L (equal to 10 mg/L nitrogen in nitrate). However, it is now thought that methemoglobinemia *per se* was not caused by nitrate itself, but by fecal bacteria that infected infants and produced NO in their gut. A recent report by Avery has argued that it is unlikely that nitrate causes methemoglobinemia without bacterial contamination, and also that the 40–50 mg/L limit on nitrate in drinking water is not necessary [43]. However, there are now legal limits to the concentrations of nitrate and nitrite in both food and drinking water. The WHO showed that the Acceptable Daily Intake for humans (ADI) for nitrate and nitrite were 3.7 and 0.07 mg/kg body weight/day, respectively, which were based on the calculations from the doses of <500 mg of sodium nitrate/kg body weight that were harmless to rats and dogs. The international estimates of nitrate intake from food are 31–185 mg/day in Europe and 40–100 mg/day in the United States [44,45]. However, the Ministry of Health, Labour and Welfare of Japan reported that the average intake of nitrate in the Japanese populations is around 200–300 mg/day, which is one and a half times to two times the ADI. Furthermore, according to a report by Hord [28], in which the daily nitrate and nitrite intakes were calculated based on the variations using the vegetable and fruit components of the DASH (Dietary Approaches to Stop Hypertension) dietary pattern [46], the level easily exceeds 1,200 mg/day nitrate. This is more than five-fold higher than the WHO’s ADI of 3.7 mg nitrate/kg body weight/day, and more than two-fold the US Environmental Protection Agency’s level of 7.0 mg nitrate/kg body weight/day for a 60 kg individual [28]. Furthermore, as indicated in Figure 2, approximately 25% of the ingested nitrate is secreted in saliva, and 20% of the secreted nitrate in saliva is converted to nitrite by commensal bacteria on the tongue [22], indicating that about 5% of the originally ingested nitrate is swallowed into the stomach (Figure 1). Therefore, for a DASH diet containing 1200 mg nitrate, an individual would be expected to swallow approximately 45 mg of nitrite a day, which easily exceeds the ADI of nitrite. Therefore, a comprehensive reevaluation of the health effects of dietary sources of nitrate/nitrite might be required in the near future [28]. Another major health concern regarding dietary nitrate/nitrite is whether dietary nitrate can cause cancer. In fact, nitrate and nitrite themselves are not carcinogenic, but nitrite which is formed from dietary nitrate might react with dietary amines to form carcinogenic nitrosoamines. This phenomenon will be discussed in detail below.

## 4. Protective Effects of Dietary Nitrate/Nitrite on Lifestyle-Related Diseases

Lifestyle-related disease is a chronic disease characterized by oxidative and proinflammatory state with reduced NO bioavailability [47]. The cellular redox balance in these patients shifts toward a more oxidizing state which affects a number of protein functions at the transcriptional and posttranslational levels, consequently disrupting the cellular homeostasis [11,40]. However, increased NO bioavailability can improve the intracellular redox environment by *S*-nitrosylation-mediated modulation of most classes of proteins present in all cells [9,40]. Recently, accumulating evidence has suggested that dietary nitrate/nitrite improves the features of lifestyle-related diseases by enhancing NO availability, and thus provides potential options for prevention and therapy for these patients [28]. Based on the recent evidence, the beneficial effects of a diet rich in these components are discussed below, focusing on insulin resistance, hypertension, cardiac ischemia/reperfusion injury, chronic obstructive pulmonary disease (COPD), cancer, and osteoporosis.

### 4.1. Insulin Resistance

**Figure 4:**
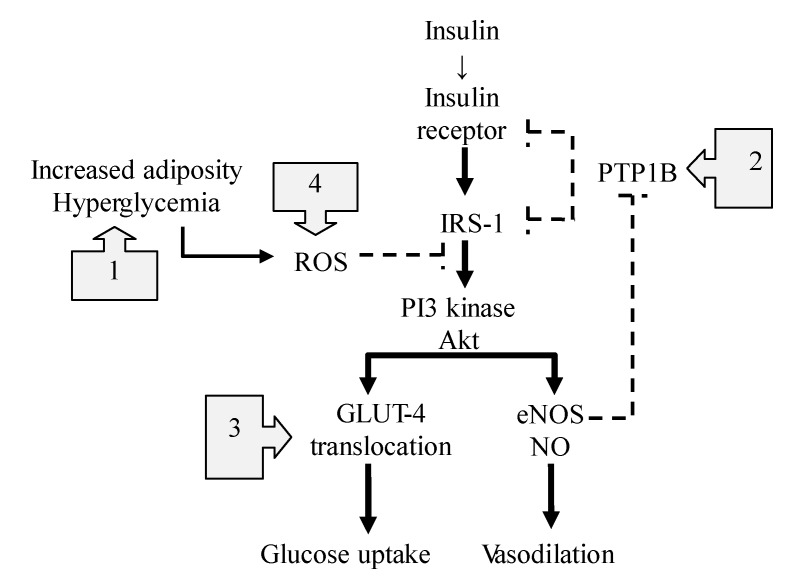
The NO-mediated actions on insulin signaling pathway. The boxes with arrows indicate the sites of NO-mediated actions against insulin resistance. Dotted lines represent inhibition, and solid lines represent stimulation. (**1**) NO suppresses TLR4-mediated inflammation and ROS production; (**2**) NO enhances the effects of insulin through the *S-*nitrosylation-mediated inhibition of phosphatase activity of PTPB1; (**3**) NO-dependent nitrosylation of GLUT4 facilitates glucose uptake; (**4**) NO inhibits mitochondrial ROS production through *S-*nitrosylation of the mitochondrial respiratory chain complex. IRS-1: insulin receptor substrate-1, ROS: reactive oxygen species, NO: nitric oxide, eNOS: endothelial NO synthase, GLUT4: glucose transporter 4, PI3 kinase: phosphatidylinositol 3-kinase, PTPB1: protein-tyrosine phosphatase B1, TLR4: toll like receptor 4.

The insulin receptor shares a signaling pathway with the activation of endothelial NOS (eNOS) [19,48,49,50,51,52] to regulate the postprandial blood flow and efficient nutrient disposition to peripheral tissues (Figure 4). Therefore, insulin resistance is always associated with impaired NO availability, suggesting that a reciprocal relationship exists between insulin activation and endothelial function [50,53]. Insulin resistance is improved by NO at various levels including insulin secretion [54,55], mitochondrial function [56], modulation of inflammation [57], insulin signaling [58] and glucose uptake [59]. For example, insulin-stimulated NO production has physiological consequences resulting in capillary recruitment and increased blood flow in skeletal muscle, leading to efficient glucose disposal [52].

However, the most important mechanism to improve insulin resistance might be at the post-receptor level of insulin signaling [60] (Figure 4). In diabetic states, increased adiposity releases free fatty acids and produces excessive reactive oxygen species (ROS) through a toll-like receptor 4 (TLR4)-mediated mechanism, which activates a number of kinases and phosphatases [61], and then disrupts the balance of protein phosphorylation/dephosphorylation associated with insulin signaling [62]. The mechanisms underlying the NO-mediated beneficial effects on insulin resistance are as follows (Figure 4): First, NO suppresses the TLR4-mediated inflammation and ROS production by inactivating IkB kinase-β/nuclear factor-κB (IκκB/NF-κβ) [9,63], the main trigger for the induction of a number of proinflammatory cytokines. Second, Wang *et al.*, indicated that NO mediates the *S-*nitrosylation of protein-tyrosine phosphatase 1B (PTPB1) and enhances the effects of insulin [52]. Because PTPB1 dephosphorylates the insulin receptor and its substrates, attenuating the insulin effect, its phosphatase activity tends to be suppressed by eNOS-mediated *S-*nitrosylation. In contrast, when the vascular eNOS activity is impaired, PTPB1 suppresses the downstream signaling to PI3K/Akt, leading to insulin resistance. Therefore, NO might act as a key regulatory mediator for the downstream signaling linking glucose transporter 4 (GLUT4) translocation and glucose uptake [58,64]. Third, Jiang recently reported that NO-dependent nitrosylation of GLUT4 facilitates GLUT4 translocation to the membrane for glucose uptake, and improves insulin resistance [65]. Fourth, excess nutrients also overproduce superoxide in the mitochondrial respiratory chain, leading to the subsequent formation of ROS. NO can inhibit mitochondrial ROS production through the *S-*nitrosylation of mitochondrial respiratory chain complex 1 enzyme and by improving the efficiency of oxidative phosphorylation in the mitochondria [12].

Indeed, the therapeutic potential of dietary nitrate/nitrite has been supported by recent studies demonstrating the improvements of insulin resistance in humans and animals as a result of its enhancing the NO availability in plasma and tissues [65,66,67,68]. As mentioned above, insulin resistance always accompanies metabolic and endothelial dysfunction, which lead to hypertension and atherosclerosis [50,51,53,69,70]. Enhancement of the availability of NO might therefore be a promising strategy for the prevention and treatment of patients with not only insulin resistance, but also endothelial dysfunction [71].

### 4.2. Hypertension

Increased consumption of fruits and vegetables is associated with a reduction of the risk of cardiovascular disease [72,73,74]. The DASH studies recommended the consumption of diets rich in vegetables and low-fat dairy products to lower blood pressure, and these effects are thought to be attributable to the high calcium, potassium, polyphenols and fiber and low sodium content in these food items [75,76]. However, vegetable diets containing high nitrate levels increase the plasma levels of nitrate and nitrite [77], which are the physiological substrates for NO production. Accumulating evidence has recently indicated that the nitrate/nitrite content of the fruits and vegetables could contribute to their cardiovascular health benefits in animals [29,33,78,79,80,81,82,83] and humans [31,84,85,86].

A number of publications have demonstrated that dietary nitrate reduces blood pressure in humans [87,88,89]. Larsen *et al.*, reported that the diastolic blood pressure in healthy volunteers was reduced by dietary sodium nitrate (at a dose of 0.1 mmol/kg body weight per day) corresponding to the amount normally found in 150 to 250 g of a nitrate-rich vegetable, such as spinach, beetroot, or lettuce [84]. Webb *et al.*, studied the blood pressure and flow-mediated dilation of healthy volunteers, and showed that the vasoprotective effects of dietary nitrate (a single dose of 500 mL of beetroot juice containing 45.0 ± 2.6 mmol/L nitrate), were attributable to the activity of nitrite converted from the ingested nitrate [86]. Kapil *et al.*, also showed a similar finding that consuming 250 mL of beetroot juice (5.5 mmol nitrate) enhanced the plasma levels of nitrite and cGMP with a consequent decrease in blood pressure in healthy volunteers, indicating that there was soluble guanylate cyclase-cGMP-mediated vasodilation following a conversion of the nitrite to bioactive NO [85]. They later presented the effects of dietary nitrate on hypertension, and showed the first evidence that daily dietary nitrate supplementation (250 mL of beetroot juice daily) for four weeks reduced the blood pressure, with improvements in the endothelial function and arterial stiffness in patients with hypertension [90]. Because arterial vascular remodeling is the major histological finding associated with aging, these vascular structural changes represent vascular wall fibrosis with increased collagen deposits and reduced elastin fibers, which result in arterial stiffening and subsequent hypertension in elderly patients. Sindler *et al.*, recently demonstrated that dietary nitrite (50 mg/L in drinking water) was effective in the treatment of vascular aging in mice, which was evidenced by a reduction of aortic pulse wave velocity and normalization of NO-mediated endothelium-dependent dilation. They showed that these improvements were mediated by reduction of oxidative stress and inflammation, which were linked to mitochondrial biogenesis and health as a result of increased dietary nitrite. These beneficial effects were also evident with dietary nitrate in their study [91], suggesting that dietary nitrate/nitrite may be useful for the prevention and treatment of chronic age-associated hypertension.

In addition, hypertension is also a major cause of ischemic heart and cardiac muscle remodeling, which lead to congestive heart failure. Bhushan *et al.*, reported that dietary nitrite supplementation in drinking water (50 mg/L sodium nitrite, for nine weeks) increased the cardiac nitrite, nitrosothiol, and cGMP levels, which improved the left ventricular function during heart failure in mice with hypertension produced by transverse aortic constriction. They also showed that dietary nitrite improved the cardiac fibrosis associated with pressure-overloaded left ventricular hypertrophy through NO-mediated cytoprotective signaling [92]. Although a number of studies on the acute effects of dietary nitrate have been conducted using animal models and healthy humans, more evidence in patients with hypertension, as well as additional studies on the long-term effects of dietary nitrate, will be needed in the future.

### 4.3. Cardiac Ischemia/Reperfusion Injury

During heart ischemia, ATP is progressively depleted in cardiac muscle cells, which impairs ion pumps, leads to the accumulation of calcium ion, and consequently damages the cell membrane stability. On reperfusion, the cardiac muscle cells are further injured, because in the mitochondria, ROS are produced in large quantities due to massive electron leaks and the formation of superoxide with the resupplied oxygen, which denatures cytosolic enzymes and destroys cell membranes by lipid peroxidation. ROS-mediated dysfunction of the sarcoplasmic reticulum also induces massive intracellular calcium overload, leading to the opening of the mitochondrial permeability transition pore and causing cell apoptosis or necrosis, depending on the intracellular ATP levels [93,94]. The availability of vascular NO would thus be expected to be impaired due to the reduced NOS activity in ischemia and subsequent consumption by superoxide during reperfusion [95], resulting in severe ischemia/reperfusion injury [30].

Nitrite, nitrate, and NO-related compounds (e.g., *S*-nitrosothiols) are constitutively present in blood and tissues. The nitrite level in cardiac tissue is a couple of times higher than that in plasma due to an unknown form of active transport from blood to tissues or due to the oxidation of endogenously generated-NO to nitrite by ceruloplasmin [96], and serves as a significant extravascular pool for NO during tissue hypoxia [97]. Carlström *et al.*, showed that dietary nitrate increased the tissue levels of nitrite and *S*-nitrosothiols in the heart, and attenuated oxidative stress and prevented cardiac injury in Sprague-Dawley rats subjected to unilateral nephrectomy and a high-salt diet [37]. Shiva *et al.*, recently showed that the nitrite stored in the heart and liver via systemic and oral routes augmented the tolerance to ischemia/reperfusion injury in the mouse heart and liver [33].

Although the genetic overexpression of eNOS in mice attenuates myocardial infarction [98], in general, the protective effects of NO on cardiac ischemia/reperfusion injury depend on the local stock of nitrite and its subsequent reduction to NO at the critical moment when NOS activity is lacking under hypoxic conditions. Indeed, the tissue levels of *S*-nitrosothiols (NO-mediated signaling molecules) are enhanced through the nitrite reduction due to NOS inhibition, hypoxia, and acidosis [97], suggesting that the tissue nitrite stores can be regarded as a backup and on-demand NO donor. There are a number of factors that have been demonstrated to reduce nitrite in the tissues, including deoxyhemoglobin, deoxymyoglobin, xanthine oxidoreductase, heme-based enzymes in the mitochondria and acidosis during ischemia [99,100]. In patients with coronary heart disease, the different consequences of myocardial infarction may depend on the patient’s daily intake of nitrate/nitrite. Indeed, Bryan *et al.*, showed that dietary nitrite (50 mg/L) or nitrate (1 g/L) supplementation in drinking water for seven days maintained higher steady-state levels of nitrite and nitroso compounds, as well as nitrosyl-heme, in mouse cardiac muscle, and these mice exhibited a smaller cardiac infarct size after ischemia/reperfusion injury compared with control mice fed a diet deficient in nitrate/nitrite for seven days. These findings suggest that this protective nitrate/nitrite may be derived at least in part from dietary sources [29].

Shiva *et al.*, demonstrated that the cytoprotective effects of nitrite on ischemia/reperfusion injury are mediated by post-translational *S*-nitrosylation of complex 1 in the mitochondrial respiratory chain, which consequently inhibits the overall mitochondrial ROS formation and apoptotic events [101]. Another possible cytoprotective effects of nitrite may be mediated by the effects of *S*-nitrosylation on the intracellular Ca^2+^ handling, which decreases Ca^2+^ entry by inhibiting L-type Ca^2+^ channels and increasing the sarcoendoplasmic reticulum (SR) Ca^2+^ uptake by activating SR Ca^2+^ transport ATPase (SERCA2a) [102]. These effects will lead to an attenuation of the increase in cytosolic Ca^2+^ during ischemia and Ca^2+^ overload during reperfusion.

Intriguingly, recent large-scale epidemiological studies reported the preventive effects of antioxidant supplementations including vitamins E, C, and beta carotene rich in fruits and vegetables on cardiovascular disease, whereas no beneficial effects were shown in other studies, and in some cases a decrease in cardiovascular protection with these supplementations was observed [103,104,105]. On the other hand, a number of epidemiological studies have shown the preventive effects of fruits and vegetables on coronary heart disease [3,4,5,6,106]. It should be noted that the consumption of an appropriate amount of fruits and vegetables, which might contain balanced doses of nitrate/nitrite and vitamins, might be more effective with regard to health maintenance and improvement than antioxidant supplementation alone.

### 4.4. Chronic Obstructive Pulmonary Disease (COPD)

COPD is considered to be a lifestyle-related disease, because long-term tobacco smoking and subsequent chronic bronchitis are causally associated with this disease [107]. Varraso *et al.*, recently reported the importance of a healthy diet in multi-interventional programs to prevent COPD [108]. They showed that high intake of whole grains, polyunsaturated fatty acids, nuts, and long chain omega-3 fats, and low intake of red/processed meats, refined grains and sugar-sweetened drinks, were associated with a lower risk of COPD in both women and men.

Because cured meats such as bacon, sausage and ham contain high doses of nitrite for preservation, antimicrobial and color fixation, epidemiological studies have demonstrated that the consumption of cured meats is positively linked to the risk of newly diagnosed COPD [109,110,111]. Nitrite generates reactive nitrogen species, which may cause nitrosative damage to the lungs, eventually leading to structural changes like emphysema [111]. This is supported by an animal study in which rats chronically exposed to 2000 and 3000 mg/L of sodium nitrite in their drinking water for two years showed distinct lung emphysema [112]. However, the dose of nitrite used in that study was 250–350 mg/kg/day, which was too high to compare with those achieved in standard human diets [113].

In fact, cured meats have been reported to generally comprise only 4.8% of the daily nitrite intake, and surprisingly, 93% of the total ingestion of nitrite is derived from saliva [114], suggesting that cured meats provide minimal contributions to the human intake of nitrite, even if they are frequently consumed. In addition, the recent nitrite levels in processed meats have been approximately 80% lower than those in the mid-1970s in the US [115]. Therefore, discussions encompassing all ingested sources of nitrite should consider whether or not the nitrite derived only from the consumption of cured meats might be responsible for the development of COPD.

On the other hand, a number of epidemiological studies have shown the beneficial effects of *n*-3 fatty acids, vitamins, fruits and vegetables on lung functions and the risk of COPD [108,116,117,118,119,120,121,122]. Although it may be difficult to isolate the specific effects of these dietary nutrients, as discussed above, the nitrate and nitrite derived from vegetables and fruits are reduced to NO, which is followed by the formation of *S*-nitrosothiols [123], rather than the formation of nitrosamines especially in the presence of reducing agents such as vitamin C and E in the stomach [28,124]. It has been shown that high dietary nitrate intake does not cause the expected elevation of the gastric nitrite concentrations or appreciable changes in the serum nitrite concentrations [125].

As mentioned above, different from the effects of the direct elevation of nitrite concentration in the plasma, the entero-salivary route of dietary nitrate/nitrite might enhance the availability of NO through the formation of *S*-nitrosothiols and its transnitrosylation to the other thiol residues of proteins, suggesting that, depending on the tissues and organs, separate metabolic pathways might exist for NO availability in this entero-salivary route. Consistent with this idea, Larsen *et al.*, recently demonstrated that acute intravenous infusion of nitrite enhanced the plasma levels of nitrite, whereas it did not affect the oxygen consumption (VO_2_) or the resting metabolic rate (RMR) in humans. Instead, dietary nitrate significantly reduced the VO_2_ and RMR by improving the mitochondrial respiratory chain function and enhancing efficient O_2_ consumption, suggesting that rather than direct nitrite infusion to enhance the plasma nitrite levels, biologically active nitrogen oxide (including the *S*-nitrosothiols produced in the stomach) might be an important molecule for the transfer of biological NO activity for cardiopulmonary function [126]. Because COPD is a state of protein-energy malnutrition due to an increased resting metabolic rate and VO_2_, the effects of dietary nitrate on the reduction of the RMR and VO_2_ might be advantageous for patients with COPD.

Whether the role of NO in COPD is protective or pathogenic depends on the origin and concentration range of NO. NO activity derived from dietary nitrate and constitutive NOS might be protective against COPD largely through the *S*-nitrosothiol-mediated mechanism including inhibition of the noncholinergic nonadrenergic nerve activity, bronchial smooth muscle relaxation, reduction of airway hyperresponsiveness, downregulation of the proinflammatory activity of T lymphocytes, and antimicrobial defense [127]. However, the deleterious effects of NO on the development of COPD might be derived from iNOS-mediated pro-inflammatory signaling [128], which is consequently (not causally) reflected by the huge amount of NO in the exhaled air of patients with COPD [129].

Recent human studies have demonstrated that dietary nitrate (beetroot juice containing approximately 200–400 mg of nitrate) improved the exercise performance and reduced blood pressure in COPD patients [130,131]. However, large-scale epidemiological evidence of the impact of nitrate is still lacking.

### 4.5. Cancer

In the stomach, swallowed nitrite is decomposed to form a variety of nitrogen compounds, including *N*-nitrosoamines [132]. In the 1950s, Magree *et al.*, first reported that *N*-nitrosodimethylamine caused malignant primary hepatic tumors in rats [133]. After this report, a number of studies followed in relation to the carcinogenic effects of *N*-nitroso compounds in animal models [134,135]. In particular, the dietary intake of red and cured meats was found to be associated with an increased risk of certain types of cancer due to the relatively large amounts of nitrite added. However, the methodological aspects have been challenged concerning the high dose of nitrosatable amines, and the physiological difference between animals and humans [136].

In the stomach, the nitrosonium ion (NO^+^) derived from nitrite can bind to thiol compounds (R-SH) and amines (especially secondary amines: R_1_-NH-R_2_), forming *S*-nitrosothiol and *N*-nitrosamine, respectively. However, while *N*-nitrosamine formation occurs even at neutral or basic pH, *S*-nitrosothiol formation tends to occur only under acidic conditions. In addition, this reaction kinetically occurs much more easily than *N*-nitrosamine formation, particularly in the presence of vitamins C and E and polyphenols, which are highly present in fruits and vegetables, which also eliminate potent nitrosating agents such as the N_2_O_3_ formed from nitrite by decomposing them to NO. This might partly explain why patients with achlorhydria and non-vegetarians eating large amounts of cured meats are at risk of developing gastric cancer [137,138,139,140,141,142].

However, this idea appears to be inconsistent with the belief that dietary nitrite is a major cause of cancer. This is because, according to the average nitrate/nitrite intake of adults in the US, most of the daily nitrate intake (around 90%) comes from vegetables, and the nitrite intake is primarily derived from recycled nitrate in the saliva (5.2–8.6 mg/day nitrite), with very little coming from cured meats (0.05–0.6 mg/day nitrite in 50g/day cured meats) and other dietary sources (0–0.7 mg/day nitrite) [136], suggesting that the entero-salivary route may be the more important source of nitrosamine exposure than exogenous intake including cured meats, that is, spitting out saliva all day long might prevent cancer development more effectively than cutting cured meats. However, recent experimental and epidemiological studies could not demonstrate a positive relationship between nitrate consumption and the risk of cancer [121,134], and the Joint Food and Agriculture Organization/World Health Organization Expert Committee on Food Additives concluded in 2008 that there was no evidence that nitrate was carcinogenic in humans. Consistent with this, recent studies have found no link between dietary nitrate and cancer [143,144].

Bradbury *et al.*, reported a large-scale study (>500,000 participants) of the associations between fruit, vegetable, or fiber consumption and the risk of cancer at 14 different sites. They showed that there was an inverse association between fruit intake and the risk of upper gastrointestinal tract and lung cancer, as well as an inverse association between fiber intake and liver cancer. The dietary intake of vegetables, as well as fruits and fiber, was inversely associated with the risk of colorectal cancer, suggesting that there is little evidence that vegetable intake is associated with the risk of any of the individual cancer sites reviewed [145].

However, chronic inflammation, including inflammatory bowel disease and *Helicobacter pylori*-induced gastritis induce inducible NOS (iNOS) and generate large quantities of NO [22,146,147], forming nitrosating and oxidant species such as N_2_O_3_ and peroxynitrite, which might cause mutagenesis through deamination, nitration of DNA, or inhibition of the DNA repair system [148,149]. Depending on the sites and amounts of NO generation, NO might represent a double-edged sword in the sense that it confers both protective and deleterious effects on cancer development [150,151].

Meta-analyses of primary and secondary cancer prevention trials of dietary antioxidant supplements, such as beta carotene, vitamins A, C, and E, showed a lack of efficacy, and on the contrary, an increased risk of mortality [104]. Although the general role of NO in carcinogenesis is complicated, and many unknown mechanisms remain to be resolved, the dietary nitrate/nitrite (at least that obtained from plant-based foods such as fruits and vegetables) have obvious inhibitory effects on cancer risk by playing some synergistic role with other nutrients in these foods.

### 4.6. Osteoporosis

Lifestyle habits, such as smoking, alcohol intake, little or no exercise, and an inadequate amount of calcium intake all influence the calcium-vitamin D metabolism [152,153,154,155] and bone mineral density, in some cases leading to osteoporosis, particularly in postmenopausal women [156]. The implications of NOS-mediated NO in the regulation of bone cell function have been well described in a number of publications [157]. For example, iNOS-induced NO production following stimulation with proinflammatory cytokines, such as interleukin 1 (IL-1) and tumor necrosis factor-α (TNF-α), inhibits bone resorption and formation, resulting in osteoporosis in patients with inflammatory diseases such as rheumatoid arthritis [158]. On the other hand, eNOS, a constitutive NO synthase, plays an important role in regulating osteoblast activity and bone formation, because eNOS knockout mice exhibit osteoporosis due to defective bone formation, and eNOS gene polymorphisms were reported to be causally linked to osteoporosis in postmenopausal women [159].

In addition, Wimalawansa *et al.*, showed that some of the beneficial effects of estrogen on bone metabolism are mediated through a NO-cGMP-mediated pathway [160], suggesting that NO donor therapy might provide a promising alternative to estrogen therapy. In this context, it has been shown that organic nitrate NO donors, such as glycerol trinitrate, isosorbide dinitrate and mononitrate all have beneficial effects on experimental and clinical osteoporosis [161,162,163], and a number of epidemiological studies also indicated that a high fruit and vegetable intake appears to have a protective effect against osteoporosis in men and pre- and postmenopausal women [164,165,166]. However, few studies have been conducted to evaluate the detailed mechanism by which inorganic nitrate/nitrite prevents osteoporosis at the molecular level, and thus further basic research will be needed for this purpose.

## 5. Conclusions

Dietary nitrate, which is provided by fruits and vegetables, can transmit NO activities in various molecular forms, including NO, nitrite, and *S*-nitrosothiols, through the entero-salivary pathway. Although the role of diet-derived NO activity in lifestyle-related diseases is complex and remains to be fully elucidated, the intake of nitrate as a nutrient in vegetables might be beneficial to human health as a result of synergistic effects with other nutrients present in vegetables, and would be recommended as a nutritional approach to the prevention and treatment of the lifestyle related diseases.
